# The radish genome database (RadishGD): an integrated information resource for radish genomics

**DOI:** 10.1093/database/baz009

**Published:** 2019-02-05

**Authors:** Hee-Ju Yu, Seunghoon Baek, Young-Joon Lee, Ara Cho, Jeong-Hwan Mun

**Affiliations:** 1 Department of Life Science, The Catholic University of Korea, Bucheon, Korea; 2 Department of Bioscience and Bioinformatics, Myongji University, Yongin, Korea

## Abstract

Radish (*Raphanus sativus* L.) is an important root vegetable crop in the family Brassicaceae, which provides diverse nutrients for human health and is closely related to the *Brassica* crop species. Recently, we sequenced and assembled the radish genome into nine chromosome pseudomolecules. In addition, we developed diverse genomic resources, including genetic maps, molecular markers, transcriptome, genome-wide methylation and variome data. In this study, we describe the radish genome database (RadishGD), including details of data sets that we generated and the web interface that allows access to these data. RadishGD comprises six major units that enable researchers and general users to search, browse and analyze the radish genomic data in an integrated manner. The Search unit provides gene structures and sequences for gene models through keyword or BLAST searches. The Genome browser displays graphic representations of gene models, mRNAs, repetitive sequences, genome-wide methylation and variomes among various genotypes. The Functional annotation unit offers gene ontology, plant ontology, pathway and gene family information for gene models. The Genetic map unit provides information about markers and their genetic locations using two types of genetic maps. The Expression unit presents transcriptional characteristics and methylation levels for each gene in 18 tissues. All sequence data incorporated into RadishGD can be downloaded from the Data resources unit. RadishGD will be continually updated to serve as a community resource for radish genomics and breeding research.

## Introduction

Rapid advances in plant genomics have enabled the development of various tools for crop improvement, including molecular markers, genetic maps, genomic data, plant resources and novel information about traits and statistics. A unified information platform integrating the available genomic data and tools would serve as a fundamental resource for research and breeding of crop species of interest. The eventual application of genomics tools and resources to agriculture includes genome-assisted selection and breeding techniques aimed at both population improvement and cultivar release (1).

Radish (*Raphanus sativus* L.) is a major root vegetable crop in the family Brassicaceae that is cultivated worldwide, serving as a source of carbohydrates, nutrients, phytochemicals and dietary fiber for human nutrition. Radish is a close relative of other *Brassica* crops, such as Chinese cabbage, cabbage, mustard and rapeseed, as well as the model plant *Arabidopsis thaliana*. The most commercially important part of the radish is its elongated, fleshy taproot. Other parts of the radish, including leaves, young siliques, seeds and seedling sprouts are also consumed. In Eastern Asia, radish has occupied an important position in the seed industry, and diverse cultivars have been developed in both public and commercial breeding programs (2). Due to its importance in agriculture, a variety of genomics studies of the cultivated radish have been performed over the last decade. A number of genetic maps have been constructed using various molecular markers (3–6). Transcriptome analyses and RNA sequencing (RNA-seq) of radish tissues have identified several genes of interest (7–11). In addition, at least four independent genome assemblies of radish cultivars have been published and these data are publicly available (12–16). Comprehensive studies of the radish genome have reported fundamental information about its genome structure and evolutionary rearrangement of chromosomes after polyploidy events, providing significant insight into the biology and breeding of this species.

Recently, we constructed a chromosome-scale genome assembly (Rs1.0) of an Asian radish cultivar, WK10039, which showed superior quality compared to other radish assemblies reported to date (7, 12, 15), in terms of coverage of the entire genome and gene space, contig number and size and anchoring of the assembly onto chromosomes (14). In addition to assembly of the draft genome (Rs1.0), we also developed a large collection of genotypes (mapping populations and germplasm accessions) and a broad array of available genomic resources such as molecular markers, genetic maps and omics data including the transcriptome, methylome and variome (5, 6, 17, 18). The data and genomic resources produced during these investigations are instrumental to understanding the unique genetic system of the radish, which has a triplicated genome structure. Moreover, these resources are beneficial for radish breeding, because they enable marker-assisted selection, comparative genomic studies and subsequent transfer of knowledge from the reference data to other radish accessions. Therefore, establishment of a web-based platform that provides sufficient information and convenient access to these genomic resources is in high demand for radish research and breeding studies.

With the aim of facilitating the use of genome assembly and other genomic information, we present herein the radish genome database (RadishGD), with details of its data sets and web interface, which allows users to fully utilize radish genomic data in an integrated manner. The first version of RadishGD (http://radish-genome.org) was made public in October 2015 and several updates have been incorporated into the current version. As of September 2018, more than 3600 cumulative database access hits with sequence downloads have been carried out by users worldwide. RadishGD comprises numerous tools, including genome assembly, chromosome-assigned pseudomolecule sequences, 46 514 protein-coding gene models, repetitive sequences, functional annotations of genes based on gene ontology (GO) and Kyoto Encyclopedia of Genes and Genomes (KEGG) pathway maps, keyword and gene ID search, genetic maps, molecular markers [conserved ortholog set (COS), insertion/deletion (InDel), simple sequence repeat (SSR) and single-nucleotide polymorphism (SNP)], Basic Local Alignment Search Tool (BLAST) sequence search and the University of California Santa Cruz (UCSC) genome browser (19) for visualization of genome sequences along with 18 transcriptomes, 8 methylomes and 17 variomes. As a fundamental genomic interface for radish biology and breeding, new genome assemblies that are constructed and relevant information provided in RadishGD will be constantly updated. Furthermore, phenotypic and sequence data about plant resources such as the core collection consisting of 125 accessions (18) will be introduced to the database in the near future.

## Data resources and analyses

### Genome assembly

For genome sequencing of the large Asian radish cultivar WK10039 (2*n* = 18 510 Mb), whole-genome shotgun sequencing was performed using a combination of the 454, Illumina, and PacBio platforms, as well as end sequencing of bacterial artificial chromosome clones with an ABI sequencer. Using ~104.4 Gb filtered sequence reads, the reference radish genome Rs1.0 was assembled into 426.2 Mb in 11 389 scaffolds consisting of 38 732 contigs through hierarchical assembly using Newbler 2.6 and Celera Assembler 7.0 (14). Rs1.0 covers 82.9% of the estimated total size of the radish genome, with a scaffold N50 of 927 kb, of which 344.0 Mb was organized into nine chromosome pseudomolecules by mapping to the reference genetic map (6).

### Repetitive sequences and gene models

The radish genome assembly was masked for class I and class II transposons with RepeatMasker (http://www.repeatmasker.org) and ~32.6% of Rs1.0 was identified as repetitive sequences. Through a combination of program-based prediction using Fgenesh+ (http://www.softberry.com), AUGUSTUS (20) and SNAP (21) with parameters trained on the radish matrix, sequence similarity-based prediction using BLAST search (22), transcriptome-based prediction using Genomic Short-read Nucleotide Alignment Program (GSNAP) (23) and Program to Assemble Spliced Alignments (24) and consensus gene structure prediction using EVidenceModeler (24), we identified a total of 46 514 protein-coding genes encoding 67 016 proteins in 84 967 transcripts. In addition, a total of 9518 RNA genes were identified using tRNAscan-SE (25) to identify transfer RNAs and BLASTN for ribosomal RNAs and micro RNAs. A General Feature Format file was generated for the predicted gene models according to the positions of genes in the genome assembly.

### Functional annotation

The protein-coding genes were annotated using the SwissProt and TrEMBL databases of UniProt (http://www.ebi.ac.uk/uniprot) with BLASTP. Motifs, domains, GO and plant ontology (PO) data were annotated using InterPro (http://www.ebi.ac.uk/interpro). The molecular interaction network among radish genes was predicted using a KEGG pathway database (http://www.genome.jp/kegg/pathway.html). Through this functional annotation, we identified 41 866 (90%) genes as ‘known’ whereas the remaining 4648 (10%) were assigned as ‘unknown’ or ‘hypothetical’. The annotations were listed as gene name, GO, PO, KEGG pathway map and Enzyme Commission number. Families of orthologous genes among radish, *A. thaliana*, *Brassica rapa* and *Brassica oleracea* were identified using OrthoMCL v2.0 (26) with all-against-all BLASTP (E value cutoff of 1E^−5^ and > 50% coverage) searches.

### Molecular markers

Two different approaches were employed to develop molecular markers based on the radish genome. First, through multiple alignment of radish single-copy genes with their counterpart COS in *Brassica* species, we identified 332 COS markers (5). Second, using genome-wide sequence comparison of the genetic mapping parents, WK10039 and WK10024, we identified 14 741 length variants and 1 258 476 SNPs, among which 882 SNP markers and 118 InDel markers were developed into non-redundant framework genetic markers for genetic mapping (6). In addition, a total of 2637 sequence-tagged site (InDel, SNP and SSR) markers were anchored to the scaffolds of Rs1.0, resulting in the assembly of chromosome pseudomolecules (14).

### Genetic map

For the purpose of constructing the radish genetic map, a reference mapping population was developed consisting of 93 F_2_ progeny from a cross between maternal WK10039 and paternal WK10024. Using this mapping population, the first radish genetic map was developed by analyzing the segregation of 322 COS markers at log of the odds (LOD) of 5.0 (5). This map was improved to create a high-density genetic map for genome sequencing through whole-genome resequencing and genotyping of the mapping population and analyses of 2637 markers at LOD 10 (6).

### Transcriptomes

To obtain a transcriptome data set, ~ 2.9 billion Illumina RNA-seq reads were generated from 18 radish tissues, including seedling (1 day, 5 days and 2 weeks after germination), leaf (4 weeks old, 10 weeks old, 10 weeks old with vernalization for 6 weeks and 11 weeks old with vernalization for 6 weeks followed by 1 week recovery), root (1–8 weeks old) and flower (petal, anther and pistil) based on three biological replicates (14, 17). The transcriptome data were aligned to the genome assembly using GSNAP (23) for gene prediction. For gene expression analyses, RNA-seq reads were aligned to the coding sequences of gene models using TopHat v2.1.0 (27) with default settings. The resulting mapped reads for each gene were normalized using Cufflinks v2.2.1 (27). The average fragments per kilobase of transcript per million mapped reads (FPKM) values for the genes were calculated and analyzed.

### Methylomes

The genomic methylation landscape of the radish was examined using single-nucleotide resolution DNA methylation maps of eight tissues, including seedling (5 days old), leaf (4 weeks old, 10 weeks old, 10 weeks old with vernalization for 6 weeks and 11 weeks old with vernalization for 6 weeks followed by 1 week recovery), root (4 weeks old) and flower (anther and pistil). To obtain a methylome data set, bisulfite conversion of genomic DNA was combined with Illumina sequencing. Bisulfite sequencing data with at least 20× coverage was generated from each tissue. The filtered reads were mapped to Rs1.0 using Bowtie2 (28) and the cytosine methylation level of the radish genome was extracted using Bismark (29).

### Variomes

Genomic variation between 10 cultivated and 7 wild radish accessions were investigated through high-depth resequencing analyses. At least 25× coverage of Illumina sequences was generated for each genotype and ~98% of the filtered reads were mapped to Rs1.0 using BWA-MEM (http://arxiv.org/pdf/1303.3997v2.pdf). Genotyping of the alignment was performed using UnifiedGenotyper in GATK (30) and a total of 4 033 566 homozygous SNPs/InDels were identified through multi-sample genotyping. Variome comparison was performed through multidimensional scaling and showed that cultivated Asian radish types are closely related to wild Asian accessions (17).

## Database features

### RadishGD structure

**Figure 1:**
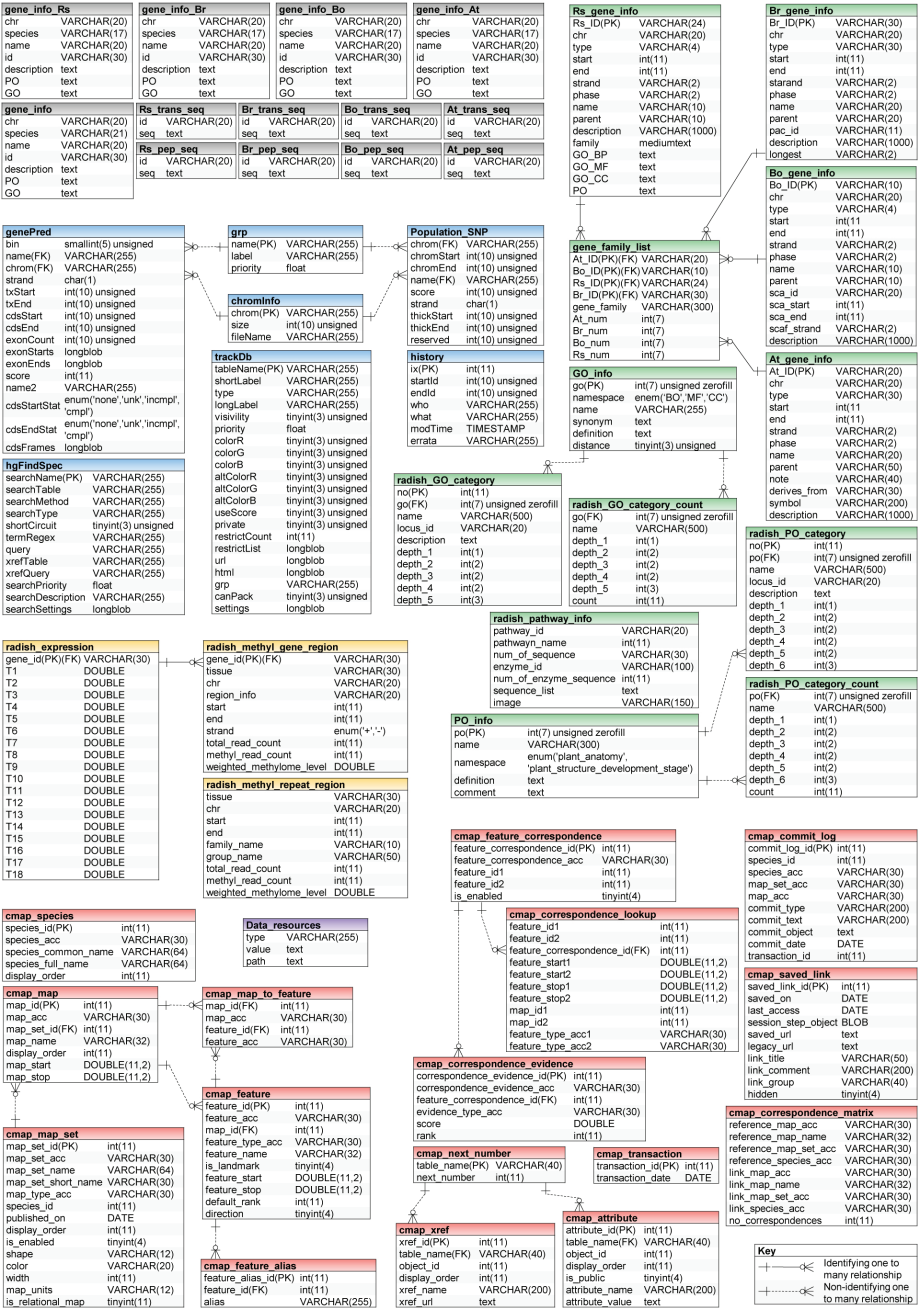
Database structure of RadishGD showing organization and relationship of entities. The boxes represent SQL database tables with table names in bold and column names in regular black. RadishGD consists of six units providing access to the radish genome data sets: Search (gray), Functional annotation (green), Genome Browser (blue), Expression (yellow), Genetic map (red) and Data resources (purple).

**Figure 2:**
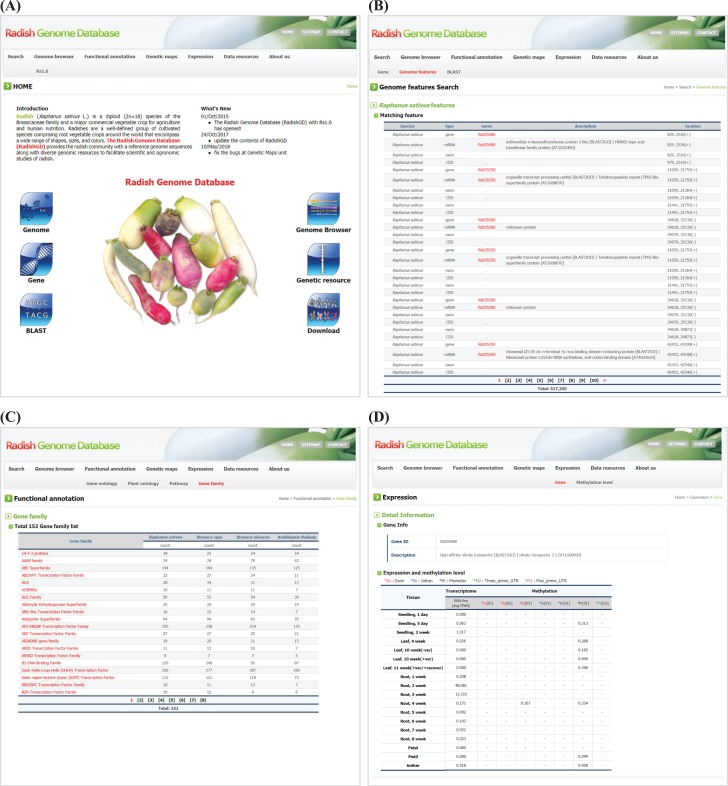
Snapshot of RadishGD home as well as ‘Search’, ‘Functional annotation’ and ‘Expression’ units providing a gene search feature, annotation and expression characteristics of protein-coding genes. (**A**) RadishGD home page at http://radish-genome.org (**B**) Access to an individual gene from genome features search. (**C**) The putative functions of gene groups predicted through gene family analysis. (**D**) Expression and methylation levels of each gene in various tissues are presented.

RadishGD was constructed using the MariaDB v.5.5.41 database engine on a server running the CentOS7 operating system. The scripting language PHP v5.4.16 was used to connect the database and web browser, and Apache v2.4.6 displayed the output of each query on the website. RadishGD integrates all of the genomic data described above. Figure 1 provides the database schema and Table 1 summarizes the genome assembly data integrated into the database. RadishGD consists of six major features used to access the radish genome data sets (Figure 2A), as described below.

### Search

RadishGD provides three major search options, ‘Gene’, ‘Genome features’ and ‘BLAST’, which are closely interconnected. The main search page, ‘Gene’, allows the user to search radish gene models as well as those of closely related species, including *A. thaliana*, *B. rapa* and *B. oleracea*, along with their sequences and annotation information. The user can identify genes of interest through keyword matching or using detailed information about the genes such as species name, chromosome number or ontology term. The search result shows a list of genes with links to detailed information about each gene provided by RadishGD for radish, TAIR (www.arabidopsis.org) for *A. thaliana*, BRAD (brassicadb.org/brad) for *B. rapa* and BolBase (ocri-genomics.org/bolbase) for *B. oleracea*. ‘Genome features’ displays the genomic location, ORF structure and sequence of each gene through a link from each gene ID (Figure 2B). ‘BLAST’ provides five BLAST search tools against *R. sativus*, *A. thaliana*, *B. rapa* and *B. oleracea* genomes using the wwwblast (31). The BLAST tools included in the Search unit correspond to the coding sequences and predicted protein sequences included in RadishGD.

### Functional annotation

To clarify the putative roles of the predicted gene models, the ‘Functional annotation’ unit categorizes genes based on their GO into molecular function, biological process and cellular component; based on their PO into plant anatomical entity, plant structure and development stage; and based on metabolic pathway mapping using the KEGG pathway database (www.kegg.jp/kegg/pathway.html). Genes are classified into each category or group on the ‘GO’, ‘PO’ and ‘KEGG pathway’ pages with links to pages providing the description, genomic position, sequence and pathway map. In addition, the ‘Gene family’ page displays a total of 152 gene families encoded in the radish, *A. thaliana*, *B. rapa* and *B. oleracea* genomes with a list of genes in each gene family and link to further information (Figure 2C). Clicking on a gene ID for a species displays the corresponding information on a new page.

**Table 1:** Summary statistics of Rs1.0 in RadishGD with functional annotation of protein-coding genes and genetic markers

Chromosome	Sequence length (bp)	No. of gene	Annotation type of gene					Genetic marker	
			GO	PO	KEGG	Gene family		SNP/InDel/SSR	COS
R1	26 309 735	3353	2104	2441	264	671		85	28
R2	43 799 612	5365	3430	4029	478	1121		141	45
R3	29 132 933	3429	2189	2448	273	635		88	28
R4	50 002 108	6146	3940	4451	468	1302		143	31
R5	45 943 323	6066	4180	4734	574	1310		129	50
R6	53 636 577	6481	4306	4859	523	1445		147	48
R7	27 187 321	3377	2268	2561	290	757		56	30
R8	29 681 327	3371	2153	2495	282	645		75	26
R9	38 354 807	4279	2740	3139	382	833		136	36
R0	82 154 559	4647	1996	2269	242	489			
Total	426 202 302	46 514	29 306	33 426	3776	9208		1000	322

R0 comprises scaffolds that have not been anchored onto nine radish chromosomes.

KEGG, KEGG pathway.

### Expression

Transcriptome and methylation data are essential to characterize the functions and expression patterns of genes. The ‘Expression’ unit enables the user to search the expression and methylation levels of each gene (Figure 2D). The transcriptome data support a total of 43 447 protein-coding gene models. On the ‘Gene’ page, clicking of a gene ID in the table displays a page showing the expression and methylation level of that gene. Otherwise, the user can search for genes of interest using the gene ID or a description of the gene. The search result includes transcription and methylation levels of the gene in 18 and 8 tissues, respectively, where transcription is presented as the average FPKM value of RNA-seq reads and methylation of each genomic region (promoter, exon, intron and untranslated regions) is given as the average cytosine methylation level. The ‘Methylation level’ page summarizes the overall methylation level of the radish genome for each tissue type. The radish genome showed an average cytosine methylation level of ~14% with relatively low methylation in vegetative tissues (10–15%) and high methylation in reproductive tissues (16–17%). In the context of cytosine methylation types, 40–55% of all CGs, 20–28% of all CHGs (H is A, T, or C) and 3–7% of all CHHs were methylated throughout the radish genome.

### Genetic maps

**Figure 3:**
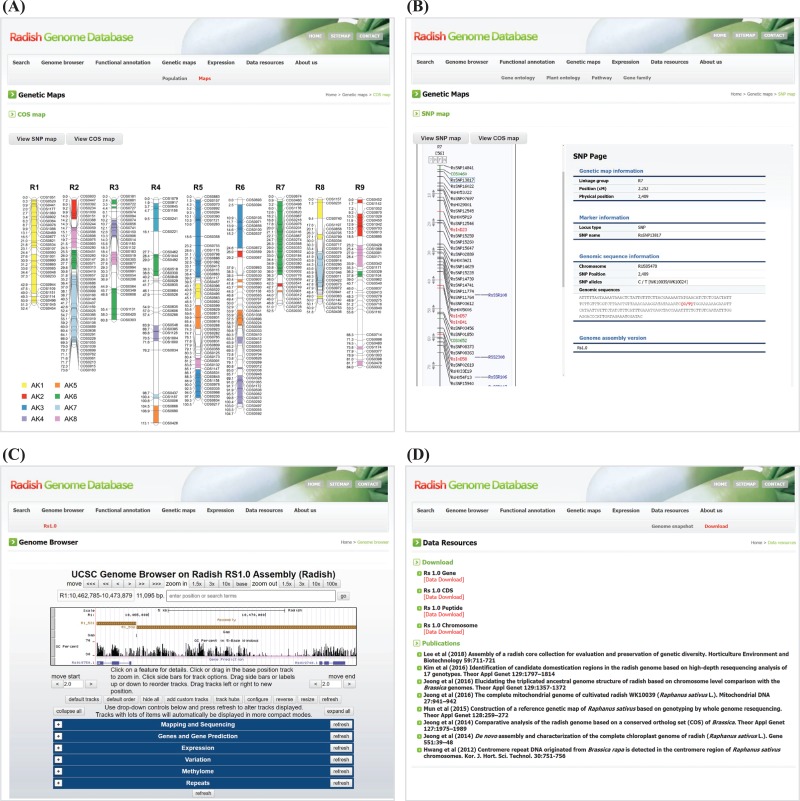
Snapshot of the ‘Genetic map’, ‘Genome browser’ and ‘Data resources’ units. (**A**) COS map providing access to molecular marker information. (**B**) Example of SNP marker on the SNP map. (**C**) The UCSC genome browser consisting of the following six tracks: read mapping and sequencing, gene models and gene predictions, expression data for 18 tissues, variation data for 17 genotypes, methylation data for 8 tissues and repeat sequence data. (**D**) Downloadable data and relevant works about radish genomics published to date are listed.

Genetic resources including mapping population, genetic maps and molecular markers are provided in the ‘Genetic maps’ unit. Genetic analyses of radish sequences were performed using an F_2_ population composed of 93 individuals that were derived from a single F_1_ plant based on a cross between cv. WK10039, the radish reference species, and cv. WK10024. The ‘Population’ page shows photographs of the parental and F_1_ plants of the mapping population. The ‘Maps’ page presents two genetic maps, the SNP map and the COS map. The SNP map was constructed from analyses of 2637 markers spanning 1538 cM with 1000 unique framework loci (882 SNP, 91 InDel and 27 SSR markers). The COS map was constructed through analyzing the segregation of 322 COS-SNP markers spanning 693.5 cM (Figure 3A). Comparative mapping of the translocated proto-Calepine karyotype-like ancestral karyotype of tribe Brassiceae using COS markers revealed the triplicated genome structure of the radish, which suggests that whole-genome triplication had occurred. Both genetic maps offer links to a marker page where the user can find each linkage group presented through the CMap viewer, along with detailed attributes of markers including marker type, genetic position and the sequences of marker loci or primers (Figure 3B).

### Genome browser

RadishGD offers access to genome sequence data integrated with a collection of all aligned annotation data sets of the radish genome using the UCSC genome browser (Figure 3C). The basic format of this display is to show the radish genome sequence in the horizontal dimension along with graphical representations of the locations of gene models, mRNAs and repetitive sequences as well as gene expression, genome-wide methylation and variation between genotypes. Presentation of the data in a graphical format allows the user to rapidly access detailed information about the annotation and facilitates examination and querying of the data. Below the displayed image in the browser, six categories of information can be selected and displayed alongside the original genomic data. These categories include mapping and sequencing, genes and gene predictions, expression, variation, methylome and repeats.

### Data resources

The ‘Data resources’ page hosts genomic data files for download (Figure 3D). The ‘Genome snapshot’ page shows overall statistics of genome assembly, predicted gene models and repetitive sequences. The ‘Download’ page offers bulk downloading of genome sequence data including chromosome-level genome assembly and the sequences of predicted genes, coding sequences and peptides. It also provides a list of literature published to date. This unit plays an important role, allowing the user to easily access radish genome sequences and literature, thereby providing contents aimed at researchers of radish biology.

### Summary and future directions

A publicly available information platform for the genome of a crop provides opportunities for comprehensive study of the plant system as well as robust application of genomics data for breeding studies. For example, fine-scale mapping and positional cloning of genes requires diverse markers and accurate genome assembly combined with the ability to browse the entire genomic context. Development of RadishGD enables integration and interconnection of all available data that we have generated through genomic study of the radish, distinguishing itself from other radish genomic databases such as *R. sativus* Genome DataBase (radish.kazusa.or.jp) and NODAI Radish genome database (www.nodai-genome-d.org), which are focused on the assembly of Japanese cultivars, and RadishBase (http://bioinfo.bti.cornell.edu/radish), which comprises the mitochondrial genome, expressed sequence tags and genetic maps of radish.

Compared to other radish databases, RadishGD provides the highest quality chromosome-level assembly of the radish genome, as well as functional annotation of genes, in-depth information about gene expression and methylation, genetic maps and marker technologies, whole-genome resequencing data and supporting literature. These data are tightly interconnected and integrated into a user-friendly platform in which genomic information is visualized from the gene to chromosome level in a ‘Genome Browser’. Therefore, RadishGD represents a valuable public resource that can be used not only to annotate and study the radish genome but also to translate the radish genome for breeding studies and pangenomic comparison with closely related Brassicaceae species.

Efforts to improve RadishGD by adding additional data and analyses are ongoing. We are currently studying radish genome sequencing and assembly based on long-read sequencing technology to improve the genome assembly. In parallel, an interface providing genome-wide information about micro RNA and long noncoding RNA is under construction. We are also developing phenotype data and whole-genome resequencing data for the radish core collection consisting of 125 accessions (18). In addition, a genome synteny browser showing the correspondence between the radish and *Brassica* genomes will be made available. Inclusion of new assemblies, phenotype data in the core collection and genome-wide SNPs associated with particular traits and activities will substantially upgrade the quality of RadishGD, resulting in continuing improvement of radish community resources. All data presented in this study and further updates are available through the RadishGD website. Therefore, the most significant beneficiaries of RadishGD will be radish researchers, including plant biologists and breeders. Increasing genomic discoveries and information resources will undoubtedly facilitate identification of novel genes and other biological studies of the radish, eventually accelerating its improvement through genome-assisted breeding.

#### Availability of database

The authors ensure the database remain available for at least 2 years following publication of the paper in DATABASE.

#### Deposition of sequence

The assembled sequence and gene models have been deposited in the National Center for Biotechnology and Information under GenBank Accession JRUI00000000.

## Notes

**Database URL**: http://radish-genome.org
